# Comparative Analysis of Convergent Jellyfish Eyes Reveals Extensive Differences in Expression of Vision‐Related Genes

**DOI:** 10.1002/ece3.71784

**Published:** 2025-07-14

**Authors:** Natasha Picciani, Cory A. Berger, Sofie Nielsen, Jacob Musser, Adam Philip Oel, Marina I. Stoilova, Detlev Arendt, Anders Garm, Todd H. Oakley

**Affiliations:** ^1^ Department of Ecology, Evolution, and Marine Biology University of California Santa Barbara California USA; ^2^ Marine Biological Section University of Copenhagen Copenhagen Denmark; ^3^ Developmental Biology Unit European Molecular Biology Laboratory Heidelberg Germany; ^4^ Department of Molecular, Cellular, and Developmental Biology Yale University New Haven USA; ^5^ Department of Ecology and Evolutionary Biology University of Kansas Lawrence Kansas USA

## Abstract

Quantifying gene expression across convergent origins of traits clarifies the degree to which those traits arise from shared versus distinct genetic programs, revealing how gene reuse relates to the repeatability of evolution. Eyes are important traits that evolved in many distantly related lineages, including at least nine times within cnidarians. Here, we investigate gene expression in eye‐bearing and nonvisual tissues from three cnidarian species representing long‐diverged lineages where eyes evolved convergently (Cubozoa, Scyphozoa, and Hydrozoa). We find gene expression in eye‐bearing tissues to be mostly lineage‐specific, with only a small proportion of genes having convergent expression across species. Nevertheless, all species express homologs of deeply conserved vision‐related genes known from Bilateria, which likely reflects deep homology (parallel evolution across vast phylogenetic distances) of a metazoan phototransduction toolkit. A gene tree analysis of opsins—the prototypical animal photosensors—shows that convergent eyes recruited different opsin paralogs, with the potential exception of an opsin ortholog shared between scyphozoan and cubozoan eyes. Our results suggest that eyes have mostly lineage‐specific patterns of gene expression, yet some key phototransduction components are repeatedly recruited across multiple independent eye origins in Medusozoa.

## Graphical abstract

Eyes have evolved in many different lineages, including several times within jellyfish, yet the relative contributions of shared and lineage‐specific genes to convergent eyes are not known. We find that three long‐diverged species of jellyfish have mostly divergent patterns of gene expression in their eye‐bearing tissues, suggesting a major role for lineage‐specific novelty in eye evolution. However, all three species also expressed homologs of bilaterian light perception genes, indicating some components of early animal phototransduction toolkits have likely been recruited into eyes repeatedly in Bilateria and Cnidaria.

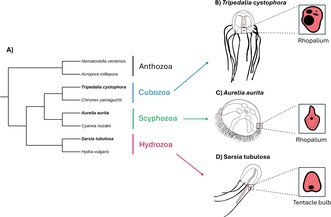

## 1 Introduction

Convergent evolution occurs when separate lineages evolve similar features, providing natural replicates that can clarify evolutionary processes and constraints underlying trait origins. Eyes, defined minimally as regions of photoreceptor cells adjacent to shading pigments (Arendt and Wittbrodt 2001), have evolved many times in animals and are an excellent system to study how evolution produces sensory structures. Eyes probably evolve in the context of pre‐existing light reception systems where photoreceptor cells first perform nondirectional light sensing and later add components such as pigments and lenses (Nilsson 2013; Oakley and Speiser 2015; Picciani et al. 2021). Thus, although eyes themselves are convergent, they may recruit homologous components of an ancestral phototransduction toolkit (Oakley 2024; Vöcking et al. 2022). This pattern is sometimes referred to as deep homology or parallel evolution (Shubin et al. 2009). However, we do not know the extent to which eyes recruit homologous genetic components and lineage‐specific novel genes because most eye origins have not been characterized at the molecular level. Eyes have evolved convergently at least nine times within medusozoan cnidarians (jellyfish), and jellyfish eyes span a range of morphologies from simple eyespots to complex lensed eyes (Berger 1898; Conant 1898; Miranda and Collins 2019; Picciani et al. 2018). Examining the multiple eye origins within Medusozoa can help us gauge the extent to which convergent morphological evolution is associated with the repeated recruitment of homologous genes versus lineage‐specific genetic solutions.

Eyes occur in each of the three major medusozoan classes: Cubozoa (one origin), Scyphozoa (one to two origins), and Hydrozoa (six or more origins; Picciani et al. 2018). There is also a probable origin within the enigmatic Staurozoa (Miranda and Collins 2019). Cubozoans, or box jellyfish, possess the most complex eyes among cnidarians and display visually guided behavior (Bielecki et al. 2023); they have true image‐forming eyes as well as other, simpler eye types (Nilsson et al. 2005). Cubozoan and scyphozoan eyes are located on sensory structures called rhopalia, which contain a large part of their nervous system and perform other sensory functions (Marques and Collins 2004; Satterlie 2011). Among hydrozoan jellyfish (“hydromedusae”), when present, eyes are located on structures called tentacle bulbs. Tentacle bulbs and rhopalia are not considered homologous structures (Marques and Collins 2004), though they may be analogous hubs of nervous and sensory functions (Denker et al. 2008; Nakanishi et al. 2009). Hydrozoan eyes span a range of complexity from simple pigment spots to lens‐bearing eyes (Picciani et al. 2018; Weber 1981). For most eye origins, we have no or very little information about their genetic basis.

Knowledge of eye‐related genes in jellyfish is currently insufficient to compare genetic similarities among eye‐bearing lineages, as only a few genes in a handful of species have been implicated in eye function and development (Ruzickova et al. 2009; Suga et al. 2010, 2008). However, both eye‐bearing and eyeless cnidarians use opsin‐based phototransduction mediated by cyclic nucleotide‐gated (CNG) ion channels, as in Bilateria (Plachetzki et al. 2010; Vöcking et al. 2022). Genes encoding components of visual phototransduction cascades (opsins, G‐proteins, and intermediary enzymes such as adenylate cyclase) and regulators of eye development (*pax*B) are expressed in box jellyfish eyes (Bielecki et al. 2014; Koyanagi et al. 2008; Kozmik et al. 2003). Knowledge of vision‐related genes in Scyphozoa is limited, although *Aurelia aurita* medusae express homologs of some bilaterian eye development genes (Gold et al. 2019). Within Hydrozoa, *Cladonema radiatum* uses *pax*A and *Six*‐family transcription factors in the development of its lens‐eyes (Stierwald et al. 2004; Suga et al. 2010), and eye‐specific opsins have also been characterized in this species (Suga et al. 2008). Various neuropeptides have been characterized in medusozoans, some of which are expressed in the rhopalia and eyes of cubozoans (Nielsen et al. 2021) and scyphozoans (Nakanishi et al. 2009). However, it remains to be seen whether these neuropeptides have specific roles in vision. Overall, genes expressed in eye tissues have not been characterized for most eye‐bearing medusozoans, preventing us from identifying shared and lineage‐specific genetic programs underlying eye evolution.

To address this limitation and determine the extent of genetic convergence among jellyfish eyes, we can use genome‐wide approaches to gain a more comprehensive perspective on vision‐related genes. Here, we compare gene expression in eye‐bearing tissues from three species spanning the major clades of Medusozoa (Cubozoa, Scyphozoa, and Hydrozoa) and conduct a phylogenetic analysis of opsins in these species. We show that eye tissues (rhopalia and tentacle bulbs) from these distantly related taxa share relatively few transcriptomic similarities, indicating that most eye‐related gene expression has evolved in lineage‐specific ways. Nevertheless, we identify some homologous genes expressed convergently in the eye‐bearing tissues of all three species, including members of gene families with highly conserved roles in bilaterian vision. Our results suggest a pattern of lineage‐specific innovation building on a conserved light reception toolkit and lay the groundwork for further comparisons across the many additional eye origins within jellyfish and other animals.

## 2 Methods

### 2.1 Animal Culturing

We cultured polyp colonies of the hydromedusa *Sarsia tubulosa* (M. Sars 1835) in a closed system with seawater at 18°C and 37‰ salinity as well as a 12:12 photoperiod. We obtained polyps of the scyphozoan *A. aurita* sp.1 (Linnaeus 1758) from the Santa Barbara Sea Center and kept them in an open water system with temperatures varying according to those on the coast of Santa Barbara, California. We fed polyps 3‐day‐old *Artemia* nauplii enriched with Selco (Self‐Emulsifying Lipid Concentrate; Brine Shrimp Direct) every 3 days. *Aurelia* jellyfish were kept in a goldfish bowl with oxygenation from an air tube creating a circular flow.

### 2.2 RNA Extraction for Tissue‐Specific Library Construction and Sequencing

**FIGURE 1:**
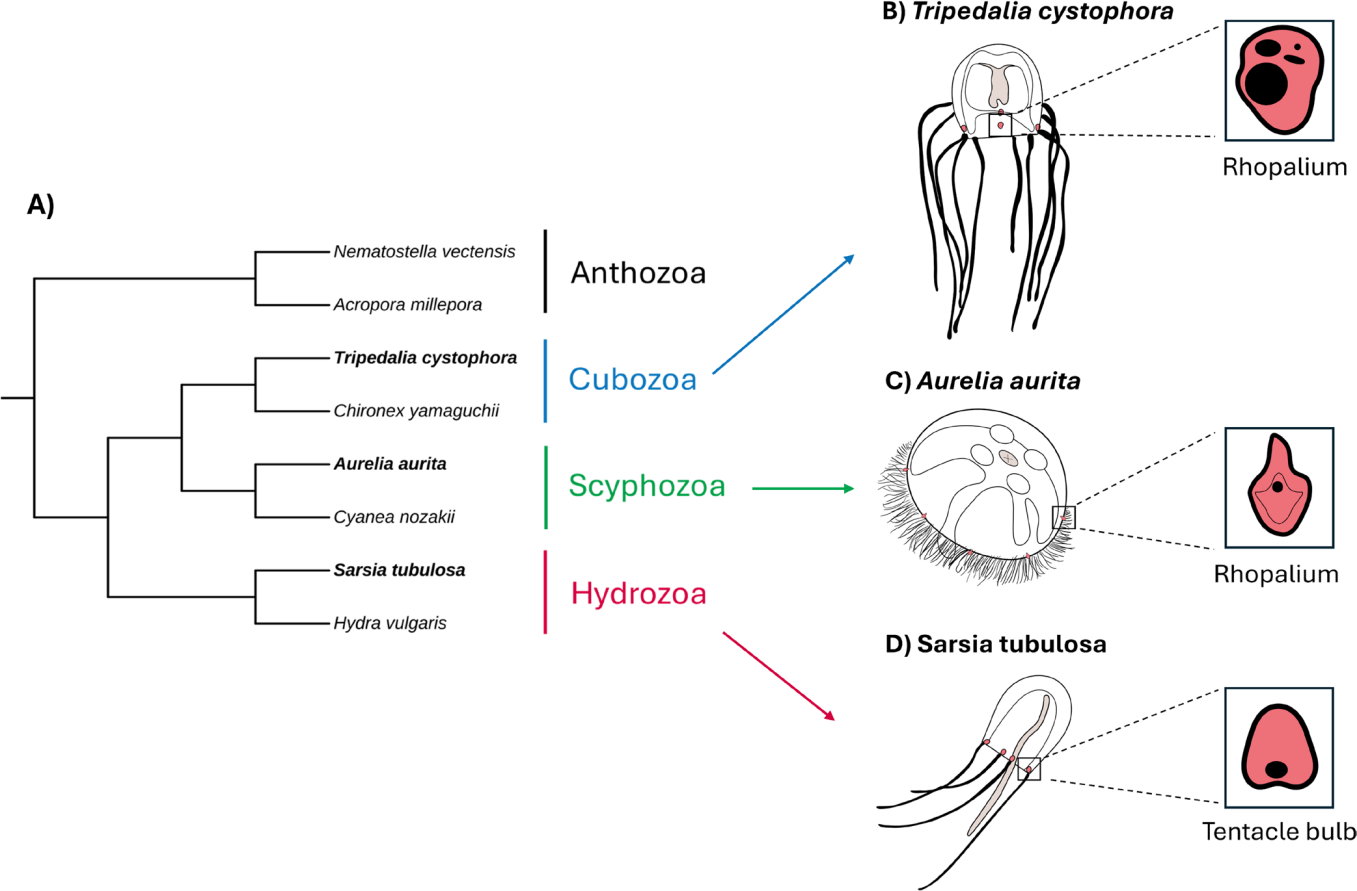
Diagrams of study species and tissue types. (A) Cladogram of study species (bold) and other species used for OrthoFinder analysis. (B–D) Sketches showing body plans of the study species highlighting the locations of eye‐bearing tissues (rhopalia and tentacle bulbs), manubria, and tentacles. Eye‐bearing tissues are colored red and shown in the inserts, with eyes represented as black ovals. Manubria (mouth and stomach) are colored cyan, and tentacles are drawn with solid black lines. Drawings are not to scale.

We starved jellyfish for at least 24 h prior to dissections in order to minimize brine shrimp contamination. We dissected tentacles, tentacle bulbs/rhopalia, and manubria (Figure 1A–C) using UV‐sterilized tools further treated with RNase AWAY Surface Decontaminant (Thermo Fisher Scientific). We transferred each dissected tissue directly into either the RNeasy Mini Kit lysis buffer or chilled TRIzol Reagent (Invitrogen) (see Table S1 for details on tissue samples and their RNA extraction methods). We proceeded according to the kit manufacturer's protocol for samples extracted with the RNeasy Mini Kit. For those kept in TRIzol, we proceeded with a liquid nitrogen freezing step followed by maceration with a mini pestle and RNA extraction using chloroform. We sent RNA samples to Novogene Corporation (Sacramento, CA) for quality control tests (quantitation and RNA integrity checks using Nanodrop, Agarose Gel electrophoresis, Agilent 2100), library preparation, and paired‐end Illumina Hiseq PE150 sequencing of 150 bp reads. We sequenced a total of 15 paired‐end RNA‐seq libraries from four tissue types from *Sarsia* and *Aurelia*, with at least two biological replicates per tissue per species (*Sarsia*, tentacles: two replicates [32 and 40 tentacles from 8 and 10 jellyfish], tentacle bulbs: two replicates [32 and 40 tentacle bulbs from 8 and 10 jellyfish], manubrium: two replicates [8 and 10 manubria from 8 and 10 jellyfish]; *Aurelia*, rhopalia: three replicates [31, 67, 67 rhopalia], and manubrium: six replicates [6, 3, 3, 9, 6, manubria; one replicate with number of manubria not recorded]). While we originally had a balanced experimental design that included three biological replicates of each tissue per species, our work was interrupted due to lab restrictions put in place at UC Santa Barbara. Additionally, we found it extremely challenging to extract high‐quality RNA from *Aurelia*; its RNA yield and quality were inconsistent despite controlled conditions and protocols. For comparative analyses, we used tissue‐specific paired‐end RNA‐seq data from tentacles (SRR8101526), rhopalia (SRR8101523), manubrium (SRR8101518), and whole medusa without tentacles, rhopalia, or manubrium (SRR8101525) of the box jellyfish *Tripedalia cystophora* Conant 1897, publicly available in the NCBI SRA database (Bioproject PRJNA498176).

### 2.3 Assembly of a Low‐Redundancy Reference Transcriptome for Sarsia

In order to ensure we captured a large diversity of expressed genes, we also extracted RNA from whole body *S. tubulosa* jellyfish individuals using the Qiagen Total RNA Kit and stranded mRNA 150 bp paired‐end sequencing with Illumina NovaSeq500. We evaluated the quality of raw reads using FastQC v0.11.9, and used Trinity v2.8.5 for assembling a combined set of this one whole body jellyfish library and three tissue‐specific libraries (one library of each tissue; tentacles, tentacle bulbs and manubrium). According to quality assessment via BUSCO v3.1 (Seppey et al. 2019), this original assembly possessed 96.3% Metazoa Complete BUSCOs, with a high proportion of duplicated BUSCOs (53.5%) among complete BUSCOs. These high levels of duplicated genes in the reference assembly led to many multimapped reads in preliminary analyses, sometimes comprising almost half of the total number of mapped reads. Multimapped reads can lead to overestimation of sequenced molecules, and, as such, are often discarded prior to differential expression analyses at the gene level (see Deschamps‐Francoeur et al. 2020). We lowered transcriptome redundancy by keeping only one transcript (the transcript coding for the longest protein) per “gene” (“gene” as defined by Trinity gene ids) following Liang et al. (2019). This nonredundant transcriptome reference had 93.4% Metazoa Complete BUSCOs and 1.9% complete and duplicate BUSCOs. We used the same strategy to build a reference assembly for *Tripedalia*, which also lacks a genome, based on its original assembly available at the NCBI TSA database (GHAQ00000000.1; Khalturin et al. 2019). By doing that, we lowered the number of sequences in our initial *Sarsia* and *Tripedalia* reference assemblies from 251,436 and 154,192 sequences, respectively, to 29,710 and 20,979. This step increased the downstream amount of uniquely mapped reads from ~40%–45% to ~75%–85%. All computing details and code used for lowering redundancy in the original references from *Sarsia* and *Tripedalia* are available at www.github.com/npicciani/eye‐expression.

### 2.4 Read Mapping and Counting

We used STAR v2.7.5b (Dobin et al. 2013) to build an index and perform read mappings of tissue‐specific libraries (see Table S2 for STAR alignment summary statistics). We counted reads mapped to genes using featureCounts v2.0.1 (Liao et al. 2014) with an annotation GTF file containing only exon entries generated with a custom python script. In order to map reads from *Aurelia*, we used the genome sequence from *A. aurita* var. Pacific Ocean published by Khalturin et al. (2019) as a reference.

### 2.5 Differential Gene Expression Analysis

We used estimated fragment counts from featureCounts to perform pairwise contrasts to identify genes differentially expressed (DE) among tissues using the R package DESeq2 (Love et al. 2014). For each pairwise contrast, the independent filtering optimizes removal of low expressed genes in order to maximize the number of rejections (Benjamini Hochberg adjusted *p*‐values lower than *α* = 0.05) over the mean of normalized counts. Each pairwise contrast was performed using Wald statistics as implemented in DESeq2, and genes were considered DE if the adjusted *p*‐value was less than 0.05. Because the *T. cystophora* samples have no biological replicates, we cannot confidently estimate differential expression. However, for exploratory purposes, we used NOISeq (Tarazona et al. 2015), which simulates technical replicates, to compare expression among *T. cystophora* libraries. For NOISeq, we used length correction (“lc = 1”) and otherwise used default parameters, and considered genes DE if they had a *q*‐value greater than 0.99. We ran NOISeq five times using different random starting seeds and found that results overlapped by ~96%, with 481/499 genes upregulated in rhopalia DE in all five runs; in this paper, we report the results with starting seed 123.

### 2.6 Orthology Assignment and Interspecies Comparisons

We used OrthoFinder v2.5.5 (Emms and Kelly 2019) to infer homology relationships. In addition to our three focal species, we provided OrthoFinder with five additional assemblies: two outgroups ( *Nematostella vectensis* jaNemVect1.1 and *Acropora millepora* Amil_v2.1) and three medusozoans belonging to the same classes as each of our three focal species: Hydrozoa (*Hydra vulgaris* PRJNA497966), Scyphozoa ( *Cyanea nozakii* SRR2089748), and Cubozoa (*Chironex yamaguchii* PRJNA498204). We did this because the inclusion of additional taxa to break up long branches can improve orthology inference in the species of interest (Emms and Kelly 2019). For the public RNA‐seq data, we assembled high‐quality transcriptomes using the following methods: we trimmed raw reads with Trimmomatic v0.39 (Bolger et al. 2014) using default parameters, performed kmer correction with RCorrector v1.0.6 (Song and Florea 2015), and removed ribosomal RNA with SortMeRNA v4.3 (Kopylova et al. 2012). We assembled transcriptomes using Trinity v2.15.1 (Grabherr et al. 2011) with default parameters and clustered transcripts at 99% identity using CD‐HIT‐EST v4.7 (Li and Godzik 2006). We predicted peptides using TransDecoder v5.7.1 (https://github.com/TransDecoder/TransDecoder), retaining peptides with hits to UniRef90 (downloaded March 8, 2024) searched by DIAMOND BLASTP v2.1.8 in “sensitive” mode (evalue = 1e‐5; Buchfink et al. 2014) or hits to PFAM (downloaded March 8, 2024) as searched by hmmscan (−E 1e‐10; http://hmmer.org). Finally, we used CD‐HIT to cluster 100% overlapping peptides (−c 1.0) and assessed transcriptome completeness with BUSCO v5.6.1 against the Metazoa odb10 database, downloaded January 11, 2024 (Simão et al. 2015). The assemblies had BUSCO scores of 94.2% ( *C. nozakii* ), 94.8% ( *H. vulgaris* ), and 95.5% (*C. yamaguchii*). We analyzed phylogenetic hierarchical orthogroups at the node corresponding to the common ancestor of the three focal species (Figure 1D). We tested the significance of overlaps in DE orthogroups with the SuperExactTest package (https://github.com/mw201608/SuperExactTest/), with the background consisting of all orthogroups with at least one gene present in all three species.

To quantify gene expression similarity, we calculated expression values within each species as TPM10K, which is similar to transcripts‐per‐million (TPM) except that it also accounts for differences in assembly size among species (Munro et al. 2022). For principal components analysis (PCA) and hierarchical clustering, TPM10K values were log‐transformed with a pseudocount of 0.001 and quantile normalized (results were similar with or without quantile normalization). PCAs were performed with the “prcomp” R function and hierarchical clustering was performed on Pearson correlations with the complete linkage method using the “heatmap.2” R function. Samples were clustered based on the set of 2682 single‐copy orthologs shared among the three species. As we initially observed strong clustering by species, we also applied batch correction following quantile normalization using ComBat in nonparametric mode (Johnson et al. 2007) and re‐performed clustering analyses. We used the “betadisper” function in the “vegan” R package to test for differences in group dispersion between eye tissues and manubria in PCA space (first 19 PC's).

### 2.7 Gene Ontology (GO) Enrichment Analysis

We performed functional annotation of all three transcriptomes using EggNOG‐mapper v2.1.12 (Cantalapiedra et al. 2021). We tested whether GO terms under the “Biological Process” category were significantly enriched among upregulated genes with the Fisher's exact test with a GO processing algorithm (“weight01”) that takes into account the hierarchical nature of GO terms using the R package topGO (Alexa et al. 2006), and a *p*‐value cutoff of 0.01. We tested the significance of GO term overlaps between species with the SuperExactTest package, with the background consisting of all GO terms found among the tested genes of all species.

### 2.8 Phylogenetic Analysis of Opsin Sequences

We retrieved opsins from *Sarsia*, *Aurelia*, and *Tripedalia* references using a customized python version of PIA (Phylogenetically Informed Annotation; Speiser et al. 2014) with the opsin tree from Picciani et al. (2018).

## 3 Results

### 3.1 Eye‐Bearing Tissues Do Not Cluster Together Across Species

We generated RNA‐seq tissue libraries for *A. aurita* (manubria and rhopalia) and *S. tubulosa* (manubria, tentacles, and tentacle bulbs), and re‐analyzed published data for *T. cystophora* (manubria, tentacles, rhopalia, and whole medusa without manubria, tentacles, and rhopalia; Bioproject PRJNA498176). The *T. cystophora* tissue libraries lacked biological replicates, and we analyzed them with simulation‐based NOISeq software (see Methods). They are included as a further point of comparison to *A. aurita* and *S. tubulosa* , but this limitation should be kept in mind.

**FIGURE 2:**
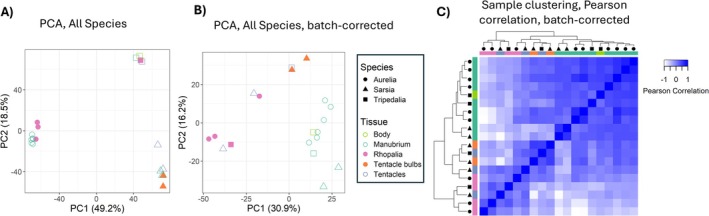
Gene expression signatures and clustering of tissue samples. (A) Principal component analysis (PCA) of tissue samples across species, calculated from expression of 2682 single‐copy orthologs with quantile normalization. Expression data were expressed as log_2_ TPM10K with a pseudocount of 0.001 and analyzed with the “prcomp” R function. Numbers in parentheses indicate the proportion of variance attributable to each principal component; filled shapes indicate eye‐bearing tissues. (B) Same as in A, except quantile‐normalized expression values were corrected for batch/species effects using ComBat. (C) Sample clustering based on Pearson correlations of samples (quantile‐normalized, batch‐corrected log_2_ TPM10k of single‐copy orthologs). Cluster dendrograms on the top and left display hierarchical clustering of tissue samples using the complete linkage method, and shapes indicate species.

Considering all species together, samples clustered strongly by species rather than tissue (Figure 2A), based on the expression of 2682 single‐copy orthologs. In a PCA, the first principal component (49% of the variance) separated *A. aurita* from the other two species, whereas the second principal component (19% of the variance) separated *S. tubulosa* and *T. cystophora* (Figure 2A). Clustering of gene expression by species rather than tissue has been observed in various invertebrates, including other cnidarians (Breschi et al. 2016; Mantica et al. 2024; Munro et al. 2022; Pankey et al. 2014). After using ComBat (Johnson et al. 2007) to remove species effects, PCAs and hierarchical clustering analysis recovered partial clustering by tissue type, particularly for manubria (Figure 2B,C). Eye‐bearing tissues did not form a single cluster, but were interspersed with tentacle samples. The *T. cystophora* rhopalia sample clustered with an *S. tubulosa* tentacle sample and two of the *A. aurita* rhopalia (Figure 2C), while *S. tubulosa* tentacle bulbs clustered with the *T. cystophora* tentacle sample.

For the batch‐corrected data, eye tissue expression was more divergent across species than manubria expression. In other words, eye tissues (rhopalia and tentacle bulbs) had greater variance in PCA space than manubria samples (betadisper, *p* = 0.0006). Thus, gene expression was less similar among likely nonhomologous tissues (rhopalia and tentacle bulbs) than tissues with likely homology (manubria), despite the convergent evolution of eyes within rhopalia and tentacle bulbs.

### 3.2 Functional Similarities Are Driven by Mostly Nonhomologous Gene Sets

To characterize gene expression signatures of eye‐bearing tissues (rhopalia and tentacle bulbs), we inferred differentially expressed genes (DEGs) between eye‐bearing tissues, manubria, and tentacles within species (Wald test, *p*_adj_ < 0.05). Table 1 lists the number of DEGs for each comparison, and Table S3 contains lists of all DEGs and their functional annotations. We subsequently focus on the comparisons between eye‐bearing tissues and manubria, and compare and contrast these results across species.

**TABLE 1:** Number of upregulated and downregulated DEGs in pairwise contrasts between tissue types in each jellyfish species (Wald test, *p*_adj_ < 0.05).

	*Sarsia*		*Aurelia*		*Tripedalia* ^a^	
	Up	Down	Up	Down	Up	Down
Rhop/TBulbs vs. Tent	1614	1815	NA	NA	359	516
Rhop/TBulbs vs. Manu	2149	1403	4339	2798	494	444
Tent vs. Manu	3192	2659	NA	NA	281	360
Total unique DEGs	7589		7137		1516	
% DEGs out of total genes	~26%		~24%		~7%	

Abbreviations: Manu, manubrium; n/a, not available; Rhop, rhopalia; Tbulbs, tentacle bulbs; Tent, tentacles.

^a^ *T. cystophora* libraries did not have replicates and were analyzed with NOISeq; these results should be viewed as exploratory. NOISeq is not deterministic and depends on the starting seed.

We identified 250 GO terms in the “biological process” category enriched among upregulated eye‐tissue DEGs in *A. aurita* , 208 in *S. tubulosa* , and 80 in *T. cystophora* . All enriched GO terms are shown in Table S4. Broadly speaking, GO terms enriched for the eye tissue vs. manubria comparison were related to cell signaling, nervous system function, and sensory processing in all species. Each species had several GO terms specifically related to light perception, such as “detection of visible light” (all three species), “phototransduction” ( *A. aurita* and *T. cystophora* ), and “visual perception” ( *A. aurita* and *S. tubulosa* ).

Overlaps of GO terms between species were significantly greater than expected by chance alone (SuperExact test, *p* << 0.01) for all pairwise comparisons and the three‐way intersection. Eleven GO terms were shared between all three species (Table 2). Eleven additional GO terms were shared between *S. tubulosa* and *T. cystophora* (including “neurotransmitter transport” and “negative regulation of calcium ion transport”), and 15 between *A. aurita* and *T. cystophora* including “phototransduction,” “cGMP‐mediated signaling,” “cAMP‐mediated signaling,” and “response to light intensity”. An additional 32 terms were shared between *A. aurita* and *S. tubulosa* , including “regulation of circadian sleep/wake cycle, non‐REM sleep,” “neuronal signal transduction,” “calcium ion transmembrane transport,” and “visual perception.” The higher number of similar GO terms in *A. aurita* and *S. tubulosa* probably reflects the fact that the *T. cystophora* analysis is exploratory and lacks statistical power.

**TABLE 2:** Overlapping GO terms from eye‐bearing tissues shared between all three species.

GO term	GO enrichment (Fisher's exact test, *p*)		
	*A. aurita*	*S. tubulosa*	*T. cystophora*
G protein‐coupled receptor signaling pathway (GO:0007186)	4.50e‐08	9.30e‐09	4.50e‐06
Regulation of cytosolic calcium ion concentration (GO:0051480)	8.40e‐06	6.57e‐03	1.86e‐03
Multicellular organismal reproductive process (GO:0048609)	6.70e‐05	3.60e‐06	4.21e‐03
Proboscis extension reflex (GO:0007637)	8.30e‐05	4.50e‐05	6.44e‐03
Motor neuron axon guidance (GO:0008045)	3.70e‐04	5.10e‐04	5.30e‐04
Positive regulation of circadian sleep/wake cycle, sleep (GO:0045938)	5.20e‐04	3.63e‐03	2.37e‐03
Detection of visible light (GO:0009584)	7.30e‐04	4.90e‐09	5.40e‐07
Sensory perception of sound (GO:0007605)	7.60e‐04	2.00e‐04	5.50e‐04
Cellular response to dopamine (GO:1903351)	1.00e‐03	3.20e‐04	1.96e‐03
Mating (GO:0007618)	1.68e‐03	6.50e‐04	5.20e‐04
Transmission of nerve impulse (GO:0019226)	2.65e‐03	7.04e‐03	1.23e‐03

As always, GO terms in nonmodel systems should not be interpreted too narrowly, but these results highlight the analogous functions of eye‐bearing tissues in these taxa, consistent with the roles of rhopalia and tentacle bulbs as centers of sensory processing. Furthermore, the enrichment of GO terms related to vision and light perception means that these jellyfish express members of gene families with known roles in bilaterian vision. The enrichment of GPCR signaling is likely also a signature of opsin‐based phototransduction, a type of GPCR signaling pathway.

Genes driving enrichment of shared GO categories across species were mostly nonhomologous. Across all GO terms shared between multiple species, an average of only 24.2% of eye‐tissue DEGs annotated with a given term were homologous—that is, they belonged to an orthogroup also containing a DEG in another species (13.9% in *A. aurita* , 24.7% in *S. tubulosa* , and 39.4% in *T. cystophora* ). Thus, functional similarities between eye‐bearing tissues are primarily driven by lineage‐specific expression of genes that are either not homologous, or that belong to large gene families split into multiple orthogroups. Nonetheless, there is a core set of homologous genes with convergent expression that contributes to a proportion of shared GO enrichment across species.

### 3.3 Convergent Gene Expression Highlights Well‐Known Vision‐Related Genes in Multiple Eye Lineages

Some genes with canonical roles in phototransduction were upregulated in the eye tissues of multiple species, notably including opsins, voltage‐gated ion channels and other components of phototransduction pathways, and transcription factors with roles in bilaterian eye development. Select DEGs discussed in this section are highlighted in Table 3, and full DEG lists can be found in Table S3. Out of 2682 single‐copy orthologs, 19 were upregulated in the eye‐bearing tissues of all three species compared to manubria. These genes included a calmodulin, a FEZ family zinc finger protein, a cytochrome P450, a dystroglycan, a Rho GTPase‐activating protein, a potassium channel, and an unannotated homeobox protein. This is a general set of genes with roles in gene regulation and signal transduction. Some of these genes have roles in the nervous systems of other metazoans (e.g., calmodulins and dystroglycans; Jahncke and Wright 2023; Solà et al. 2001), and homeobox genes function in eye development (along with development of many other structures) in both invertebrates and vertebrates.

**TABLE 3:** Select DEGs upregulated in multiple species with potential vision‐related functions.

Gene annotation	Orthogroup	No. of copies upregulated in *A. aurita*	No. of copies upregulated in *S*. *tubulosa*	No. of copies upregulated in *T. cystophora*
Adenylate cyclase	N2.HOG0005603	0	1	1
Aquaporin	N2.HOG0001516	1	0	1
CNG channel	N2.HOG0004772	4	1	1
Cyclic phosphodiesterase	N2.HOG0007678	1	1	2
Cyclin‐dependant kinase activator	N2.HOG0012444	1	1	0
Guanylate cyclase	N2.HOG0002109	6	0	1
Guanylate cyclase	N2.HOG0007504	3	1	0
Lens‐fiber membrane intrinsic protein	N2.HOG0011628	0	1	1
Lens‐fiber membrane intrinsic protein	N2.HOG0015120	0	2	1
Lhx homeobox	N2.HOG0009315	1	1	0
NR2E‐like nuclear receptor	N2.HOG0004145	1	1	0
Opsin	N2.HOG0000953	3	10	4
Opsin	N2.HOG0000854	2	0	5
Six3/6 transcription factor	N2.HOG0003095	3	1	1
SOX transcription factor	N2.HOG0001869	0	1	1
SOX transcription factor	N2.HOG0001871	1	1	0

Nine additional orthologs were upregulated in the rhopalia of *A. aurita* and *T. cystophora* , including a putative neuropeptide receptor, a voltage‐dependent calcium channel subunit, and a protein kinase. A further 27 genes were upregulated in *A. aurita* rhopalia and *S. tubulosa* tentacle bulbs, including several calcium, sodium, and potassium channels; a hippocalcin‐like protein (which in vertebrates is a neuron‐specific calcium‐binding protein); a calsyntenin; and additional transcription factors. Finally, six more orthologs were shared between *S. tubulosa* and *T. cystophora* , including an adenylate cyclase, a Kelch‐like protein, and a (non‐opsin) GPCR.

Extending our analysis from single‐copy genes to members of the same orthogroup (which can be orthologs or paralogs), we found 25 multicopy orthogroups containing at least one gene upregulated in eye tissues of all three species. Additionally, 49 multicopy orthogroups were shared between *A. aurita* and *T. cystophora* , 82 between *A. aurita* and *S. tubulosa* , and 49 between *S. tubulosa* and *T. cystophora* . The numbers of overlapping DE orthogroups were no greater than expected by chance given the number of shared orthogroups between the three species (SuperExact test, *p* = 0.16). In *A. aurita, S. tubulosa*, and *T. cystophora* , 6%, 11%, and 37% of DEGs belonged to an orthogroup with at least one DEG in another species, respectively (including single‐copy orthologs).

Among the orthogroups shared in all three species, there were a few genes with potential roles in phototransduction or eye development: several opsins, a CNG ion channel, and a six3/6 (optix) homeobox gene (Table 3). There were also numerous other transcription factors of various families, several GPCRs with similarity to neurotransmitter or hormone receptors, and a 5′‐cyclic phosphodiesterase. Multicopy genes shared between *A. aurita* and *T. cystophora* included additional opsins, a nuclear receptor, guanylate cyclases, and aquaporins, while those shared between *S. tubulosa* and *T. cystophora* included a SOX transcription factor, a guanylate cyclase, and a lens fiber membrane intrinsic protein (Lmip). Multicopy genes shared between *A. aurita* and *S. tubulosa* included additional calcium and potassium channels, calcium‐binding proteins, GPCRs, SOX transcription factors, a nuclear receptor similar to a vertebrate photoreceptor‐specific NR (NR2E), guanylate cyclases, and Lhx homeobox proteins.

Finally, we examined potential vision‐related genes that were only DE in a single species. In *A. aurita* , this includes additional ion channels, two cGMP‐dependent phosphodiesterases, a G‐protein, a regulator of G‐protein signaling gene (RGS), and a Wnt gene. *T. cystophora* rhopalia upregulated three crystallins (two of which were previously characterized as J1 lens eye crystallins in this species; Piatigorsky et al. 1993), a gene similar to a retinal homeobox protein Rax, another CNG channel, and a homolog of a bone‐morphogenic protein (BMP). Lastly, *S. tubulosa* tentacle bulbs upregulated another possible Lmip gene.

### 3.4 Neuropeptides Are Broadly Expressed Across Tissue Types

Six neuropeptide candidates have been annotated from the transcriptome of *T. cystophora* (Nielsen et al. 2019), three of which (RFamide, RAamide, and VWamide) are expressed in various neuronal subpopulations both within and outside the rhopalia (Nielsen et al. 2021). We investigated whether the remaining neuropeptides in *T. cystophora* , along with neuropeptides in *S. tubulosa* and *A. aurita* , are also expressed in eye‐bearing tissues based on existing annotations in these species (Koch and Grimmelikhuijzen 2019; Nielsen et al. 2019). For *A. aurita* , Koch and Grimmelikhuijzen (2019) used a different reference transcriptome, so we BLASTed those sequences against our assembly. We identified three of six *A. aurita* neuropeptides among our protein‐coding genes. All three investigated *A. aurita* neuropeptides were greatly upregulated in rhopalia—although they were also highly expressed in manubria. In *T. cystophora* , the six neuropeptides were highly expressed in all tissues and had by far the highest expression in rhopalia; three (LWamide, RAamide, and FRamide) were DE. Of the nine *S. tubulosa* neuropeptides investigated, two (LWamide and WLKGRFamide) were upregulated in tentacle bulbs compared to manubria, but both also had high expression in tentacles. RPRamide and FFPRamide were upregulated in manubria.

### 3.5 Opsins Expressed in Eye‐Bearing Tissues Are Orthologs of Known Visual Opsins

**FIGURE 3:**
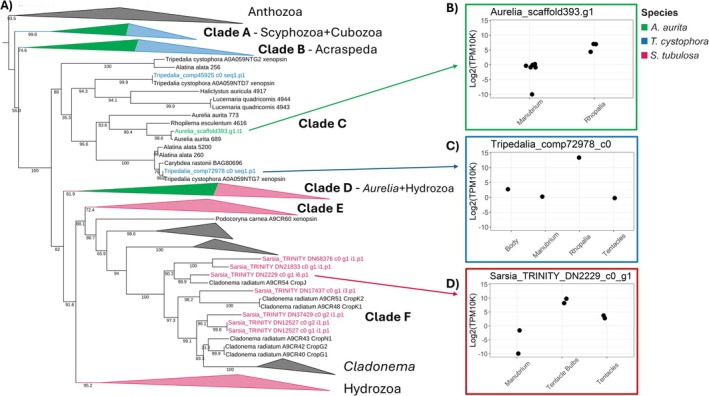
Cnidops gene tree (A) and expression of selected opsin genes (B–D). Maximum likelihood tree inferred from a sequence alignment of anthozoan and medusozoan “cnidops” sequences under the JTT + F + R8 model in IQ‐TREE. Ultrafast bootstrap support values calculated from 1000 replicates shown above branches. Clades are collapsed for visualization, with groups containing opsins from *A. aurita* , *T. cystophora* , and *S. tubulosa* colored green, blue, and pink, respectively. Clade labels are referenced in the main text, and the full tree is in the Supporting Information.

Because opsins are the quintessential light sensors in animal eyes, we investigated the expression levels and evolution of this gene family. Using phylogenetically informed annotation (PIA; Speiser et al. 2014) and manual inspection of BLAST hits, we identified 6, 28, and 20 candidate opsins in *A. aurita* , *S. tubulosa* , and *T. cystophora* , respectively. In the latter two species, several of these were partial‐length sequences lacking the lysine residue (K296) to which the photosensitive chromophore attaches (Hargrave et al. 1983). These short sequences were excluded from further analysis, resulting in final sets of 25 and 13 opsins in *S. tubulosa* and *T. cystophora* , respectively. Consistent with previous studies, we found that opsin genes from each species all belong to the cnidops clade (Figure 3A), the only type of opsins known from medusozoans (Picciani et al. 2018).

Medusozoan visual opsins have been characterized in the lens eyes of *T. cystophora* (comp72978_c0; referred to as lens‐eye opsin [LEO] in Bielecki et al. 2014)) and in the eyes of the hydromedusa *C. radiatum* (Suga et al. 2008). Expression of all opsins in our transcriptomes is shown in Table S5. The LEO had by far the highest expression in the *T. cystophora* dataset, with expression 4 orders of magnitude higher in rhopalia than manubria. In total, 10/13 *T. cystophora* opsins were DE, all of which had dramatically higher expression in rhopalia than manubria (Table S5); most of these had low or negligible expression in manubria and tentacles, including even the three opsins that did not meet the statistical criteria for differential expression. These results were consistent with localization of opsins by antibody staining (Garm et al. 2022): the LEO and another eye‐specific opsin, the “slit‐eye opsin” (comp45925_c0), were both only expressed in rhopalia. In *A. aurita* , four of six investigated opsins were DE, all of which had higher expression in the rhopalia and low‐to‐negligible expression in manubria (Table S5). In the gene tree, *A. aurita* opsins fell into three groups: nested in a clade with scyphozoan and staurozoan sequences (Clades A and B, Figure 3A); sister to another scyphozoan opsin in a clade with cubozoan opsins, including the LEO of *Tripedalia* (Clade C); and in a clade with hydrozoan sequences (Clade D). The opsin orthologous to the LEO of *T. cystophora* had the second‐highest rhopalia expression of the *A. aurita* opsins (Figure 3B, Table S5).

In *S. tubulosa* , patterns of opsin expression across tissues were more variable (Table S5). Four opsins had high tentacle bulb‐specific expression (upregulated in tentacle bulbs compared to both manubria and tentacles), while four were upregulated in both tentacle bulbs and tentacles. Two opsins had the highest expression in manubria, and one had the highest expression in tentacles. Thus, in *S. tubulosa* , not all opsins are associated with eye‐bearing tissues, whereas all *A. aurita* and *T. cystophora* opsins were primarily expressed in rhopalia.

Strikingly, the *S. tubulosa* opsin with the strongest tentacle bulb‐specific expression was sister to the CropJ eye opsin from *C. radiatum* (Clade F, Figure 3A,D). Two other *S. tubulosa* opsins are also closely related to CropJ: one of these was upregulated exclusively in tentacle bulbs, and another was upregulated in both tentacle bulbs and tentacles (Clade F). CropK1 and CropK2 are other opsins expressed specifically in the ocelli of *C. radiatum* ; the *S. tubulosa* ortholog of CropK1/K2 also had tentacle bulb‐specific expression. Thus, there is a clade of opsins in *S. tubulosa* and *C. radiatum* that have strong eye‐specific expression in *C. radiatum* and tentacle bulb‐specific expression in *S. tubulosa* (Clade F). *Cladonema* and *Sarsia* belong to separate families, but ancestral state reconstructions suggest that their eyes are homologous (Picciani et al. 2018). The observation that opsin orthologs have strong tentacle‐bulb/eye‐specific expression in both species provides further evidence for the homology of eyes in these two hydrozoan families. A clade of *C. radiatum* opsins with expression in other body parts nested within eye‐related opsins (Clade F) does not have orthologs in *S. tubulosa* . A separate clade of *S. tubulosa* opsins forms a clade with *C. radiatum* opsins that have tentacle‐specific expression (Suga et al. 2008); these were all either non‐DE or had high expression in both tentacles and tentacle bulbs (Clade E). This clade of opsins thus does not appear to have eye‐specific expression but is expressed more broadly in tentacles and tentacle bulbs in both species. The remaining *S. tubulosa* opsins, some of which were highly expressed in the manubria or were not DE, were closely related to other hydrozoan sequences but did not have orthologs in *C. radiatum* .

## 4 Discussion

Separate eye origins in moon jellyfish ( *Aurelia aurita* ), box jellyfish ( *T. cystophora* ), and hydromedusae ( *S. tubulosa* ) are associated with the expression of some familiar animal eye gene families. This suggests that deeply conserved components of an ancestral phototransduction toolkit might play functional roles in convergently evolved eyes. However, we lack functional information about many of these genes in Medusozoa, and spatial expression analyses are needed to determine their specific localization within eyes rather than surrounding tissues. In addition, the vast majority of gene expression in eye‐bearing tissues is lineage‐specific. Across species, gene expression was more dissimilar among eye‐bearing tissues than among manubria, which is thought to be a homologous tissue (Hejnol and Martindale 2008; Kraus et al. 2015). These preliminary results suggest some level of convergence among eye‐bearing jellyfish tissues involving the repeated expression of known phototransduction genes, against a backdrop of extensive differences consistent with the phylogenetically independent origins of eyes. Follow‐up work with improved sample sizes and quantification of cell‐specific gene expression will be important to validate specific findings and key genes (particularly for *T. cystophora* ), which were generated without biological replicates in another study (Bioproject PRJNA498176).

Our results highlight the roles of cubozoan and scyphozoan rhopalia and hydrozoan tentacle bulbs as important components of the nervous and sensory systems, being enriched for genes related to nervous system function and sensory processing. Functional similarities (defined by shared GO terms) were greater than expected by chance alone, suggesting that eye‐bearing rhopalia and tentacle bulbs are enriched for similar molecular functions including light sensing. Nevertheless, gene expression is largely divergent between species, and eye‐bearing tissues do not clearly cluster together based on gene expression. Instead, both rhopalia and tentacle bulbs have some transcriptomic similarities with tentacles, likely reflecting their developmental origins (Figure 2; Denker et al. 2008; Nakanishi et al. 2009). Limited clustering by tissue may also reflect the enormous evolutionary distances between Scyphozoa, Cubozoa, and Hydrozoa, which diverged at least 505 Mya (Cartwright and Collins 2007). Rhopalia of *A. aurita* and *T. cystophora* appear to be more similar in gene expression to one another than to *S. tubulosa* tentacle bulbs, consistent with the evolutionary relationships of these species and the likely homology of rhopalia themselves (Marques and Collins 2004). These similarities could be driven by genes with roles in the cubozoan/scyphozoan nervous system or nonvisual sensory structures, such as sensory ciliated epithelia (Nielsen et al. 2019; Parkefelt and Ekström 2009). However, we did observe numerous examples of convergent gene expression between rhopalia and tentacle bulbs; since rhopalia and tentacle bulbs are not homologous tissues, these genes represent genuine cases of convergence in eye‐bearing structures rather than shared ancestry or ontogeny.

### 4.1 Convergent Gene Expression Identifies Candidate Genes With Roles in Phototransduction and Eye Development

Although the major signature of gene expression in our study was species‐specific, we identified numerous homologous genes expressed in eye‐bearing tissues across three distantly‐related jellyfish lineages, many of which have putative functions related to sensory processing, nervous system development, and cell signaling. Several of these genes are intriguing candidates for roles in medusozoan photoreception and eye development because they are homologs of known bilaterian vision‐related genes, including opsins, transcription factors, and lens components (Table 3).

Overlapping gene sets expressed in convergently evolved tissues could arise due to chance alone and need not invoke any particular evolutionary mechanism (e.g., Foster et al. 2022). Consistent with this, we found that overlaps of orthogroups upregulated in eye tissues were not greater than expected by chance given the number of shared orthogroups between the three species. However, our analysis probably underestimates similarities among species due to low sample sizes (particularly for *T. cystophora* ). Additionally, many convergent genes in this study are homologous to vision‐related genes from vertebrates and insects, suggesting that evolution repeatedly favors the use of these genes in eyes. These genes could represent convergence at a molecular level, in which members of the same gene family independently gained functions related to visual systems. Examples of this type of convergence could be certain transcription factors or lens‐related genes with roles in eye morphogenesis and development.

Alternatively, genes may belong to an ancestral toolkit that forms a component of eyes—a pattern of deep homology. This is supported by our observation that all three jellyfish eye tissues express homologs of canonical phototransduction genes, including opsins, CNG channels, guanylate cyclases, and cGMP‐dependent phosphodiesterases (Table 3). Additionally, there was a strong enrichment of ion channels and calcium‐binding genes (e.g., calmodulins) in all three species. Calcium signaling is a fundamental feature of bilaterian phototransduction (Koch and Dell'Orco 2013; Nakatani et al. 2002), and this also appears to be the case in cnidarians. These results suggest that ancient phototransduction genes with conserved light‐sensing functions have been repeatedly recruited into eyes (Picciani et al. 2021).

#### 4.1.1 Opsins

The expression patterns and phylogenetic relationships of opsin proteins support a pattern of lineage‐specific recruitment of visual opsins, with one possible exception in Scyphozoa and Cubozoa. We found that most *A. aurita* opsins were not closely related to box jellyfish opsins, indicating that scyphozoan and cubozoan eyes use largely different opsin complements. However, one sequence (“scaffold393.g1”) was orthologous to the known LEO of *T. cystophora* . This gene was sister to an opsin sequence from the eyeless scyphozoan *Rhopilema esculentum* , and these scyphozoan genes are orthologs of the eye‐specific opsins from box jellyfish (Figure 3A). If this gene has a role related to the eye‐spots of *A. aurita* , it may represent a case of parallel recruitment of an opsin ortholog into independently evolved eyes. An alternative scenario is that this opsin is expressed in the rhopalia but not specifically in the eyes (in which case it may also be expressed in rhopalia of eyeless scyphozoans). The opsin trees from Picciani et al. (2018) and the current study (Figure 3A) also contain *A. aurita* sequences from other published assemblies, whose expression we could not quantify here. The use of more complete transcriptome and genome assemblies may clarify the full opsin complement of *A. aurita* . At the same time, *A. aurita* is actually a cryptic species complex (Dawson and Martin 2001; Scorrano et al. 2017), so sequences from different assemblies may belong to different species.

Overall, we find that *S. tubulosa* and hydrozoans in general have a greater diversity of opsins than scyphozoans and cubozoans, and that opsins largely diversified separately in Hydrozoa and Acraspeda (Scyphozoa + Cubozoa + Staurozoa). Nearly all examined opsins in the scyphozoan *A. aurita* and the cubozoan *T. cystophora* had strong rhopalia‐specific expression. In contrast, the hydrozoan *S. tubulosa* possesses a higher number of opsins, some of which were expressed specifically in manubria, tentacles, tentacle bulbs, or in both tentacles and tentacle bulbs. This shows that opsins may have diverse tissue‐specific functions in hydrozoans, which may include light‐mediated processes such as gonad release (Quiroga Artigas et al. 2018) and modulation of cnidocyte firing (Picciani et al. 2021; Plachetzki et al. 2012), or functions unrelated to light perception.

Notably, the four *S. tubulosa* opsins with strong tentacle bulb‐specific expression are orthologs of *C. radiatum* visual opsins, suggesting that the common ancestor of these species already possessed eyes expressing two to three opsins. The *S. tubulosa* opsin with the highest expression in tentacle bulbs is an ortholog of the *C. radiatum* opsin CropJ, which is localized near the retina margin in that species (Suga et al. 2008). Overall, similarities in expression patterns of opsin orthologs provide functional evidence for the homology of eyes in these species and insights into the possible opsin complement of their ancestor, although their expression has yet to be localized specifically to eyes in *S. tubulosa* . Further work to sequence and quantify expression of opsins in other related species whose eyes are not definitively homologous (e.g., *Moerisia* spp. and *Sphaerocoryne* spp.) could clarify the number of eye origins in this group.

#### 4.1.2 Other Eye‐Related Genes

Genes encoding lens proteins were upregulated in *T. cystophora* and *S. tubulosa* . While the former species has true lenses, the ocelli of *S. tubulosa* instead contain an electron dense material that may simply provide structure rather than act as a lens (Singla and Weber 1982). We found that *T. cystophora* rhopalia upregulated two previously characterized crystallin genes (J1A and J1B; Piatigorsky et al. 1993, Piatigorsky et al. 1989), as well as an *alpha(B)‐crystallin*‐like heat‐shock protein. Crystallins are a functional class of proteins involved in lens formation and light focusing, and different eye origins usually recruit different crystallins (Oakley 2024). Crystallins are not known from other cnidarians, and rather than crystallin genes, we retrieved two lens fiber membrane intrinsic protein‐like (Lmip‐like) genes upregulated in *S. tubulosa* tentacle bulbs. Interestingly, one of the Lmip‐like genes was also upregulated in *T. cystophora* rhopalia. Lmip genes encode structural constituents of mammalian eye lenses (Alcala et al. 1975), making these *S. tubulosa* and *T. cystophora* genes intriguing candidates for the convergent recruitment of homologous genes as structural components of eyes. Finally, aquaporins were expressed in rhopalia of *A. aurita* and *T. cystophora* . Aquaporins occur in all animals and are essential components of vertebrate ocular tissues (Schey et al. 2014), again making this gene family a candidate for convergent recruitment into separate eye origins.

We also identified transcription factors related to genes with known roles in bilaterian eye development: six3/6 (optix) homeobox genes and NR2E‐like nuclear receptors expressed in all three species, Lhx1 homeobox genes expressed in *A. aurita* and *S. tubulosa* , and a retinal homeobox gene (Rax) expressed in *T. cystophora* . Six3/6 genes regulate eye development in flies and vertebrates (Anderson et al. 2012) and six1/2 and six3/6 are also expressed in *C. radiatum* eyes (Stierwald et al. 2004). Nuclear receptor subfamily 2 group E genes (NR2E) are essential for vision in other animals (Forrest and Swaroop 2012; Kitambi and Hauptmann 2007; Yu et al. 2000), and Lhx1 regulates retinal development and circadian neurogenesis in vertebrates (Bedont et al. 2014; Kawaue et al. 2012). Finally, Rax is another homeobox gene involved in vertebrate eye formation (Rodgers et al. 2018).

Box jellyfish may use eye‐specific neuropeptides as neurotransmitters, and some neuropeptides are expressed in specific neuronal subpopulations in *T. cystophora* rhopalia (Nielsen et al. 2021). However, we found that across all three species, neuropeptides were highly expressed in all tissues, suggesting a more general role in nervous system function. This is consistent with prior studies showing RFamide‐expressing neurons throughout the medusa—although other neuropeptides may have tissue‐specific expression (Nielsen et al. 2021). In *A. aurita* and *T. cystophora* , all investigated neuropeptide genes had the highest expression in rhopalia but were also highly expressed in manubria. This likely reflects the density of neurons in rhopalia rather than a specific role in visual processing. In contrast, the tentacle bulbs of *S. tubulosa* showed less of an enrichment for neuropeptides. Some *S. tubulosa* neuropeptides were most highly expressed in manubria or tentacles rather than tentacle bulbs, again suggesting functions beyond vision.

## 5 Conclusions

Many genes upregulated in eye‐bearing tissues in jellyfish are homologs of bilaterian gene families involved in vision, suggesting that cnidarian and bilaterian eyes draw from a shared genetic toolkit to some extent. This likely reflects the deep homology of animal phototransduction systems, as we observed genes related to opsin‐CNG phototransduction (e.g., opsins, ion channels, calcium‐binding proteins) upregulated in the eye tissues of multiple jellyfish species. Further characterization of ocular and nonocular phototransduction in Medusozoa is needed to determine which genes had roles in light perception that predate eyes, and which have specifically been co‐opted into eyes themselves. We also identify several key genes that may affect eye function and development. For instance, six3/6 family transcription factors were upregulated in the eye‐bearing tissues of all three species; these genes are expressed in the eyes of another hydrozoan ( *C. radiatum* ) and are also involved in eye development in *D. melanogaster* and vertebrates, making this gene family an intriguing candidate for functional testing. However, tissues bearing convergently evolved eyes in different jellyfish classes (Scyphozoa, Cubozoa, and Hydrozoa) were largely distinct transcriptomically, indicating that most eye‐related gene expression is lineage‐specific. Some level of convergence at the gene level is expected by chance due to the finite size of genomes, which may explain some of the overlapping gene sets expressed in these species. On the other hand, genes that are repeatedly expressed in vertebrate, arthropod, and cnidarian eyes may be influenced by functional constraints or evolutionary dynamics that favor their use in eyes. Eye evolution seems to involve many possible genetic solutions, but draws on some key phototransduction genes and transcription factors that are repeatedly recruited across independent eye origins. As evolutionary biologists gain more empirical knowledge about gene reuse in convergent systems, they will better understand how factors like divergence time and genetic constraints impact the repeatability of evolution.

## Author Contributions

**Natasha Picciani:** conceptualization (lead), data curation (lead), formal analysis (equal), investigation (equal), software (equal), writing – original draft (lead). **Cory A. Berger:** formal analysis (equal), investigation (equal), software (equal), visualization (lead), writing – review and editing (lead). **Sofie Nielsen:** formal analysis (supporting), investigation (supporting), writing – original draft (equal). **Jacob Musser:** data curation (equal). **Adam Philip Oel:** data curation (equal). **Marina I. Stoilova:** data curation (equal). **Detlev Arendt:** data curation (equal). **Anders Garm:** data curation (equal), writing – review and editing (supporting). **Todd H. Oakley:** conceptualization (equal), funding acquisition (lead), supervision (lead), writing – review and editing (supporting).

## Conflicts of Interest

The authors declare no conflicts of interest.

## Supporting information


**Table S1.** Details of tissue samples and RNA extractions.

**Table S2.** STAR alignment summary statistics.

**Table S3.** Differentially expressed genes and annotations.

**Table S4.** Enriched gene ontology terms.

**Table S5.** Expression of opsin genes.

## Data Availability

Raw RNA‐seq data have been uploaded to the NCBI Sequence Read Archive (SRA), Bioproject PRJNA1236395. Data files and code are deposited in the Dryad repository https://doi.org/10.5061/dryad.bvq83bknd and also available on GitHub at https://github.com/npicciani/eye‐expression.
